# Anti-leishmanial activity of Brevinin 2R and its Lauric acid conjugate type against *L. major*: *In vitro* mechanism of actions and *in vivo* treatment potentials

**DOI:** 10.1371/journal.pntd.0007217

**Published:** 2019-02-27

**Authors:** Farnaz Zahedifard, Hyeryon Lee, Joo Hwan No, Mona Salimi, Negar Seyed, Ahmad Asoodeh, Sima Rafati

**Affiliations:** 1 Immunotherapy and *Leishmania* Vaccine Research Department, Pasteur Institute of Iran, Tehran, Iran; 2 *Leishmania* Research Lab, Institut Pasteur Korea, Seongnam-si, Republic of Korea; 3 Physiology and Pharmacology Department, Pasteur Institute of Iran, Tehran, Iran; 4 Department of Chemistry, Faculty of Science, Ferdowsi University of Mashhad, Mashhad, Iran; Ohio State University, UNITED STATES

## Abstract

Leishmaniasis, as a major health problem in tropical and sub-tropical areas in the world, needs novel, safe, nontoxic and plausible therapeutic solutions for its control. As a part of innate immune system, natural antimicrobial peptides have a potential to be used as new generation of antibiotics especially after persistent resistance of conventional antimicrobial agents. Brevinin 2R, a member of Defensin families of host defense peptides, showed promising effects against bacterial and fungal infections as well as cancerous cell lines. In the current research, the anti-leishmanial effect of Brevinin 2R and its lauric acid conjugate was investigated against *Leishmania major* (*L*. *major*) parasite. The data revealed that, conjugation of fatty acid to Brevinin 2R, strengthen its effect on *L*. *major* promastigotes as well as toxicity and hemolytic effect. These peptides showed anitleishmanial activity through cell membrane disruption and changes in the electrical and mitochondrial membrane potential. No signs of apoptosis induction or caspase activation were detected. Despite its hemolytic and cytotoxic effect in *in vitro* conditions, lauric acid- Brevinin 2R (L- Brevinin 2R) did not show site specific adverse reactions in animal model. Treatment course with L- Brevinin 2R in the *L*. *major* infected mice exhibited decreased parasite load in the lymph nodes adjacent to the infected site despite cytokine production profile and footpad swelling data.

## Author summary

Seeking novel drugs against leishmaniasis is a necessity due to inefficiency of current medications. Brevinin 2R, as a non-hemolytic natural antimicrobial peptide, was effective against vast majority of bacterial and fungal infections as well as cancerous cell lines. In this regard in the current study, the efficacy of Brevinin 2R and its lauric acid conjugate version were studied against *L*. *major* parasite growth inhibition at *in vitro* and in animal model. The results exhibited that, conjugation of fatty acid to Brevinin 2R exacerbated anti-leishmanial effect. L- Brevinin 2R resolved the promastigotes through membrane disruption and changes in the membrane and mitochondrial potential. Also, L- Brevinin 2R was able to limit successfully the parasite load in the lymph nodes of *L*. *major* infected animals.

## Introduction

Leishmaniasis is a public health problem in countries of tropical and subtropical continents all over the world. Every year about 700,000 to 1 million new cases and 20,000 to 30,000 deaths are reported. Extensive climate changes in recent decades lead to global warming, provided opportunities for vector borne diseases to spread and find new territories in the world community [1]. Leishmaniasis has different clinical manifestations, from a self-healed cutaneous wound to malformed mucocutaneous nasal cavity injuries and the visceral form which is life threatening [2]. Currently, treatment of leishmaniasis as a neglected tropical disease confronts serious difficulties. As a low income community problem, leishmaniasis has not been the objective of extensive investments of pharmaceutical companies [3]. Antimonials, the oldest known medication for the disease, not only has hepatic and cardio toxicity but also has established resistance through years of routine utilization [4]. Repurposing FDA approved drugs, created new chances to introduce anti-leishmanial agents to low income communities. Amphotericine B (AmB), as an antifungal drug, in its liposomal form (Ambisome) was found effective in limiting *Leishmania* infection especially in visceral form [5]. Although with reduced renal toxicity, patient’s hospitalization and high cost of treatment overcome advantages of this novel medication [6]. Miltefosine, originally developed as an anticancer drug, is the other known therapeutic agent which was successfully tested against cutaneous infection with oral route of administration. However, its long half-life in the body and the teratogenicity cause resistance and make it unsuitable for pregnant women [7]. In this regard, there is a report of relapsing the disease in VL patients treated with miltefosine in Indian subcontinent [8]. Moreover, the mechanism of resistance generation was completely recognized by laboratory settings [9,10]. The other anti-leishmanial drug, Paromomycin, which is classified as aminoglycoside antibiotics, may cause nausea, abdominal cramps and diarrhea in patients [11]. Due to this issues, exploring the novel, low cost and safe drugs for leishmaniasis treatment are always in demand.

Among new antibiotic candidates, antimicrobial peptides (AMPs) are suggested to be promising agents against microbial infections. Regarding to innate immune system, natural AMPs are widespread in prokaryotes and eukaryotes all over the world. AMPs affect microbes either by invading to the membrane of the cell or limiting the vital functions inside the cell. In other words, they disrupt cell membrane by pore formation or preventing its construction and repair. This disruption changes the cell membrane permeability, which in turn leads to disturb cell integrity and then cell death. AMPs can activate apoptosis through inhibition of DNA, RNA or protein synthesis. Also, they are able to change mitochondrial charge and evacuate the cell from its energy source [12]. Other than direct killing, they are the oldest well-known natural immunomodulators, which can initiate several immune response cascades to protect against different threats including bacteria, fungi, protozoa and so on [12]. In recent years, several AMPs were examined against different species of *Leishmania* both *in vitro* as well as animal models. Among them, a synthetic peptide originated from Cystatin showed favorable effects against *L*. *donovani* infection in an experimental model [13]. Other types of AMPs like Urocortin II [14] and Vasoactive Intestinal peptide (VIP) analogs, were capable of controlling infection in the cutaneous form of the disease in Balb/c mice [15]. Recently, our data about HNP1 from Defensin superfamily of AMPs also showed remarkable effect on limiting *L*. *major* infection in mice model [16].

Brevinin 2R from Defensin superfamily is a cationic AMP isolated from skin secretion of green tree frog, *Rana ridibunda*. This peptide is the only non-hemolytic known member in this family, which limits different cancerous cell line proliferation [17] as well as Gram negative / positive bacteria and fungi [18]. Furthermore, Brevinin 2R exhibited immunomodulatory effect on HepG2 cancerous cells by induction of interleukin-6 (IL-6) and IL-1β expression [19]. Regarding the intracellular nature of *Leishmania* infection and based on the examples of conventional anticancer (miltefosine) or antifungal (AmB) drugs repositioned for leishmaniasis treatments, Brevinin 2R was selected to evaluate its therapeutic potential against *L*. *major* infection. Also, according to previous experiments by Chicharro C. *et al*. with lauric acid conjugation to Cecropin–Melitin hybrid peptide [20], N-terminal lauric acid conjugated form of Brevinin 2R (L—Brevinin) was entered to the research to potentiate peptide’s penetrance into the parasite membrane. Herein, the anti-leishmanial activity of Brevinin 2R and its lauric acid conjugate type against promastigote and amastigote form of *L*. *major* were assessed.

Cytotoxicity properties of peptides were tested against Tamm-Horsfall Protein 1 (THP1) cell line as well as human red blood cells (RBCs). The selected peptide along with CpG motif was tested as a therapeutic agent against experimental cutaneous leishmaniasis in Balb/c mice model. We found that, L- Brevinin 2R controlled promastigote and probably amastigote growth in the culture. Although the peptides showed cytotoxic effect against THP1 and RBCs, no site specific adverse effect was observed in mouse and the parasite load in infected animal was controlled by peptide administration. Furthermore, based on the mechanism study, L- Brevinin 2R was found to act through membrane disruption and changes in parasite mitochondrial and membrane potential.

## Results

### Antibacterial effect of Brevinin 2R and L- Brevinin 2R

One of the basic features of antimicrobial peptides is their antibacterial activities. To do it, antibacterial properties of peptides were tested against a standard species of *E*. *coli* (ATCC 25922) following their synthesis (Table 1).

**Table 1:** Peptides’ list and sequences.

**Peptide name**	**Sequence**
**Brevinin 2R**	KLKNFAKGVAQSLLNKASCKLSGQC
**L- Brevinin 2R**	lauric acid- KLKNFAKGVAQSLLNKASCKLSGQC
CLIP*	LPKPPKPVSKMRMATPLLMQALPM
**L- CLIP**	lauric acid- LPKPPKPVSKMRMATPLLMQALPM

* MHC Class II associated invariant chain peptide

In the case of Brevinin 2R, the peptide could completely inhibit bacterial growth at the concentration of 6.25 μg/ml. However, lauric acid conjugated version of the peptide showed the antibacterial effect at the lower concentrations than 6.25 μg/ml (Table 2) implying that, lauric acid conjugation increased the antibacterial activity. Interestingly, lauric acid alone couldn’t inhibit the bacterial growth at 1.56 μg/ml or even at the concentrations much more than that (1.56–50 μg/ml). These findings showed that, the combination of lauric acid and Brevinin 2R created a new spatial structure that enhanced the antibacterial effect. CLIP, as a negative control peptide, had no impact on bacterial proliferation. But, the L- CLIP, unexpectedly worked as well as L- Brevinin 2R and inhibited the bacterial colony formation at <1.56 μg/ml (Table 2). Besides, ampicillin was applied as control positive antibiotic and showed growth inhibition at higher concentrations in compare to peptides (Table 3).

**Table 2:** Antibacterial effect of different peptides and lauric acid on *E*. *coli* (ATCC 25922).

**Peptides**	**Viability of *E*. *coli* colony (%)**			
	12.5 μg/ml	6.25 μg/ml	3.12 μg/ml	1.56 μg/ml
**Brevinin 2R**	0 ± 0	0 ± 0	4.75 ± 3.18	100 ± 0
**L- Brevinin 2R**	0 ± 0	0 ± 0	0 ± 0	0 ± 0
**CLIP**	100 ± 0	100 ± 0	100 ± 0	100 ± 0
**L- CLIP**	0 ± 0	0 ± 0	0 ± 0	0 ± 0
**Lauric acid**	100 ± 0	100 ± 0	100 ± 0	100 ± 0

Table shows the percent of viable remaining bacteria, means the percent of colonies exposed to peptides in compare to negative control colony count.

**Table 3:** Percentage of viable remaining colonies of *E*. *coli* at different concentrations of ampicillin as positive control antibiotic.

**Viability of *E*. *coli* colony (%)**					
**Ampicillin Concentration**	**1600 μg/ml**	**800 μg/ml**	**400 μg/ml**	**200 μg/ml**	**100 μg/ml**
	2.9 ± 0.4	1.17 ± 1.65	2.63 ± 1.24	9.67 ± 2.90	12.3 ± 3.31

### Anti-promastigote activity of Brevinin 2R and L- Brevinin 2R

**Fig 1:**
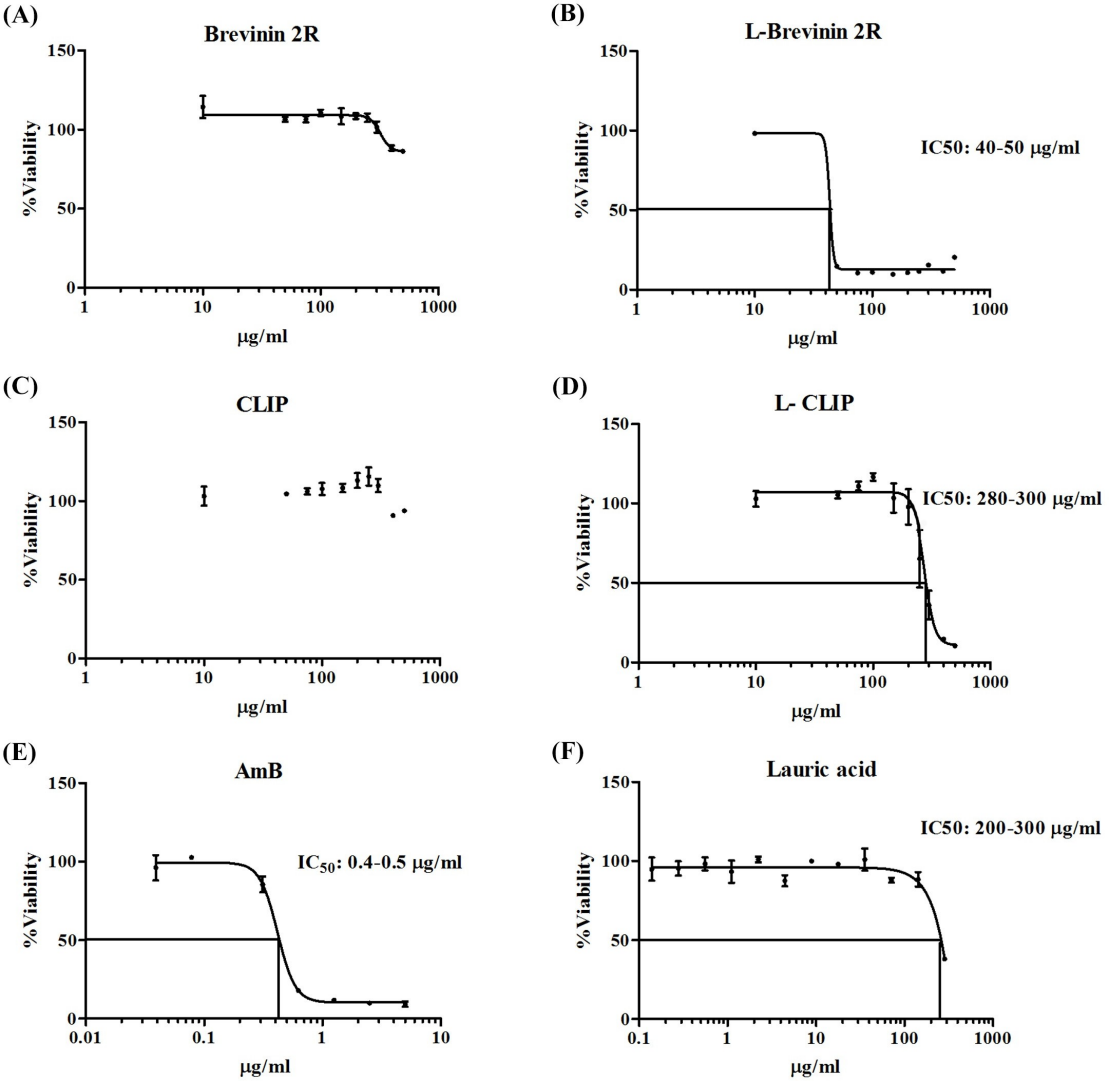
Effect of (A) Brevinin 2R, (B) L- Brevinin 2R, (C) CLIP, (D) L-CLIP, (E) AmB and (F) Lauric acid on *L*. *major* promastigote growth.

In order to evaluate the anti-leishmanial activity of these peptides, growth inhibition assay was carried out using the extracellular form of parasite, promastigotes. Brevinin 2R partially inhibited the promastigote growth at the highest applied concentration (500 μg/ml, ~ 30%). On the other hand, L- Brevinin 2R inhibited *L*. *major* promastigote proliferation with the half maximal inhibitory concentration (IC_50_) between 40 to 50 μg/ml showing a ten-fold increase in potency. Indeed, in our study the lauric acid conjugate form of CLIP (L-CLIP) was also used as negative control. CLIP as a negative control peptide did not limit the promastigote growth using different concentrations; however, L- CLIP surprisingly exhibited the antimicrobial property with IC_50_ of 280–300 μg/ml. Thus, lauric acid alone showed inhibition against promastigotes only at the concentrations more than 300 μg/ml, which demonstrated that, conjugation is an approach to increase the efficiency of this peptide (Fig 1).

### Anti-amastigote activity of Brevinin 2R and L-Brevinin 2R

**Fig 2:**
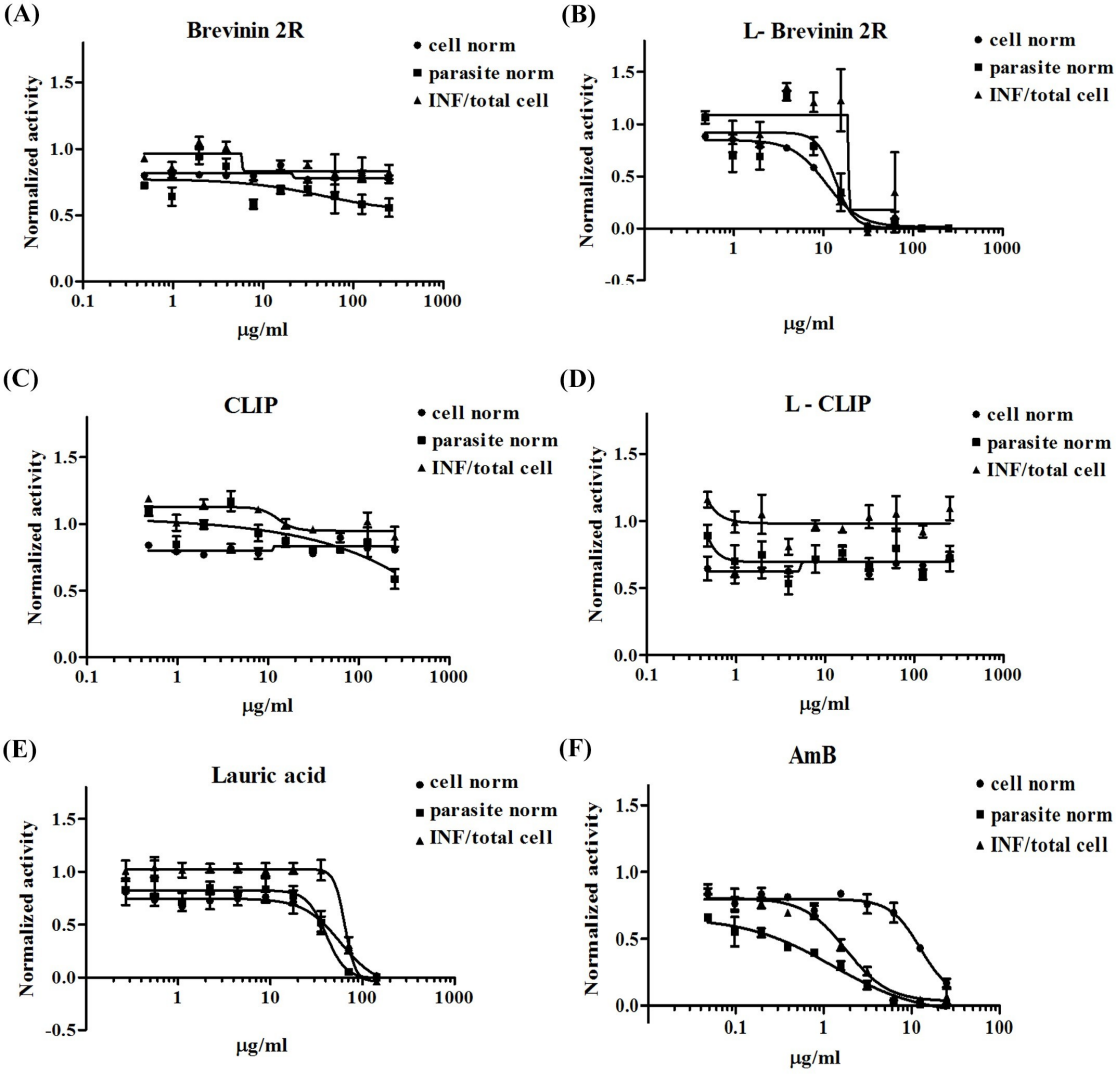
Effect of (A) Brevinin 2R, (B) L-Brevinin 2R, (C) CLIP, (D) L-CLIP, (E) Lauric acid, and (F) AmB on *L*. *major* amastigote growth and their toxicity effect on THP1 cells. Internalized promastigotes were transformed to amastigote in THP1 cells. Imaging system captured different views of the cells exposed to different concentrations of peptides. Eventually the ratio of infected to total THP1 cells (INF ratio), number of parasites presented inside the cells and number of THP1 cells were calculated.

Considering the results of promastigote growth inhibition, we further evaluated the anti-amastigote activity of mentioned peptides using *L*. *major* infected THP1 system. Ratio of infected THP1 cells, number of amastigotes and number of THP1 cells were calculated, normalized and analyzed by an image based assay system. Brevinin 2R was neither toxic against THP1 cells nor limited amastigote inside the cells. On the other hand, L- Brevinin 2R displayed toxicity to THP1 cells and also, limited amastigote proliferation at the same range of concentration (10–20 μg/ml). Due to low selectivity index of L-Brevinin 2R, the anti-amastigote activity of peptide needs further investigation. CLIP and L- CLIP as controls, did not show any effect on either THP1 cells or amastigotes, whereas lauric acid at the highest tested concentration had toxic effect on both THP1 and amastigote. The effective concentration (142.5 μg/ml) for lauric acid was far beyond L- Brevinin 2R, suggesting that, the effect of L- Brevinin 2R on amastigotes and THP1 cells was due to conjugated form of this peptide (Fig 2).

### Assessment of hemolytic activity of Brevinin 2R and L- Brevinin 2R

**Fig 3:**
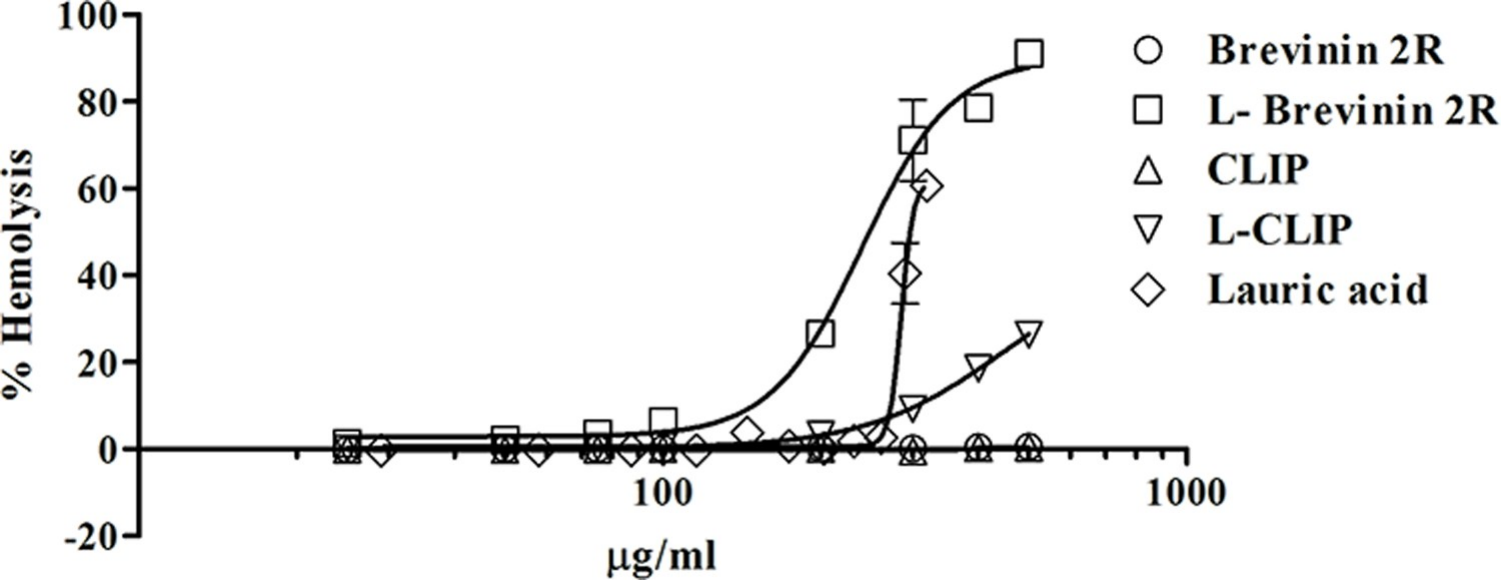
Percentage of hemolysis on human RBCs in the presence of different concentrations of peptides and lauric acid.

One of the major benefits of Brevinin 2R is its non- hemolytic activity [17]. In agreement with this note, Brevinin 2R exhibited no effect on RBCs hemolysis even at the highest tested concentration (500μg/ml). Notably, L- Brevinin 2R displayed a concentration- dependent hemolytic response with the half maximal effective concentration (EC_50_) value of 250 μg/ml. Moreover, CLIP peptide displayed no hemolytic activity, whereas L- CLIP showed about 30% hemolysis at the highest concentration. It’s worth mentioning that, lauric acid showed hemolysis (58%) of RBCs at the highest applied concentration (319 μg/ml), which collectively suggests that, none specific hemolytic activity of the conjugated peptide may be attributed to the presence of lauric acid (Fig 3).

### Increased membrane permeability by L-Brevinin 2R

**Fig 4:**
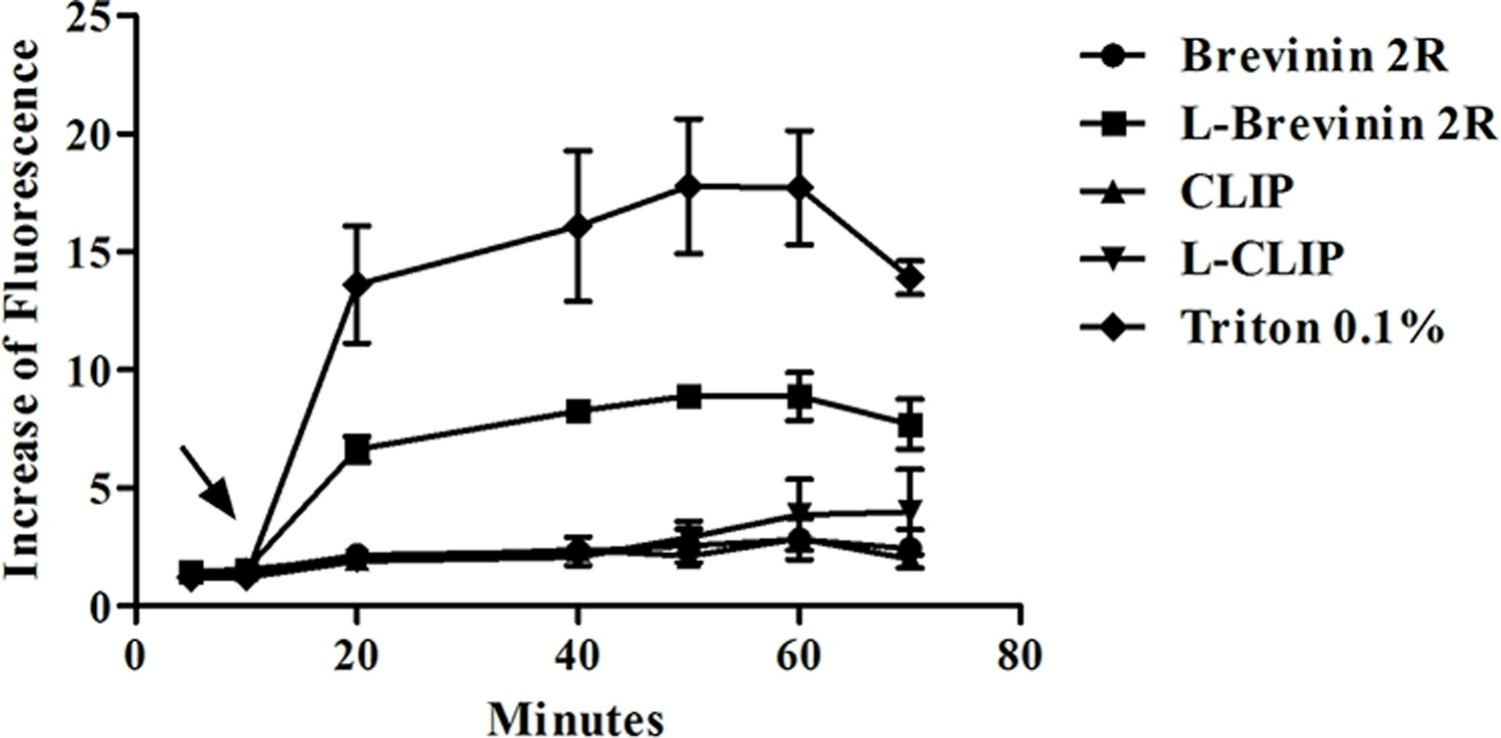
*L*. *major* promastigote permeability assay with Sytox green. Arrow shows the time of reagent addition. Increase in fluorescence intensity was calculated from the ratio of each sample to promastigote alone plus Sytox green.

Sytox green fluorescence probe penetrates inside the cell through the pore formation or complete disruption of the cell membrane. In other words, the rate of Sytox green access to the DNA, indicated the severity and strength of membrane disruption. In our experiment, after addition of peptides to promastigotes, L- Brevinin 2R immediately showed an increase in the fluorescent intensity, which was more than that of Brevinin 2R, CLIP, L- CLIP or parasite alone. In this regard, fluorescence intensity was sharply elevated following 0.1% Triton addition as a positive control. In the case of L- CLIP, as shown in Fig 4, a slight increase in fluorescence intensity was observed after 70 minutes of exposure to the peptide, which was not significant referring to CLIP as a negative control (Fig 4).

### Cell membrane potential was disturbed by L-Brevinin 2R

**Fig 5:**
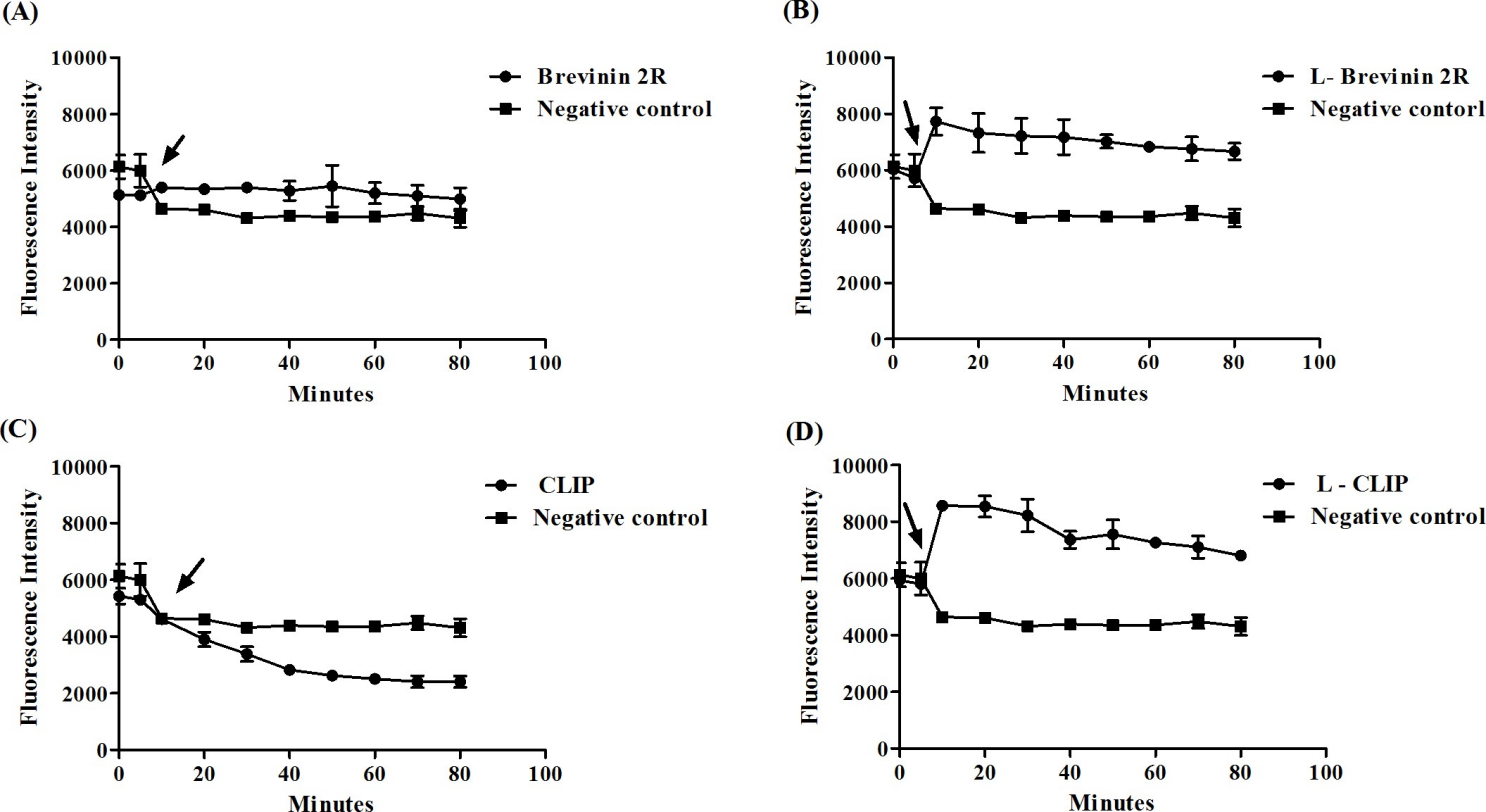
Cell membrane potential changes in *L*. *major* promastigotes exposed to (A) Brevinin 2R, (B) L- Brevinin 2R (C) CLIP and (D) L-CLIP. Arrow shows the time of peptides addition.

DiSBAC2 as a voltage sensitive probe, is able to enter depolarized cells and bind to membrane or intracellular proteins, which in turn, changes the fluorescence intensity [21]. *L*. *major* promastigotes that were exposed to L- Brevinin 2R showed a fast increase in the amount of fluorescence intensity, which was significant compared to negative control. Remarkably, using Brevinin 2R or CLIP caused no significant changes in the Fluorescence intensity; however, L- CLIP similar to L- Brevinin 2R affected cell membrane potential (Fig 5).

### Necrosis of *L*. *major* promastigotes induced by L- Brevinin 2R

**Fig 6:**
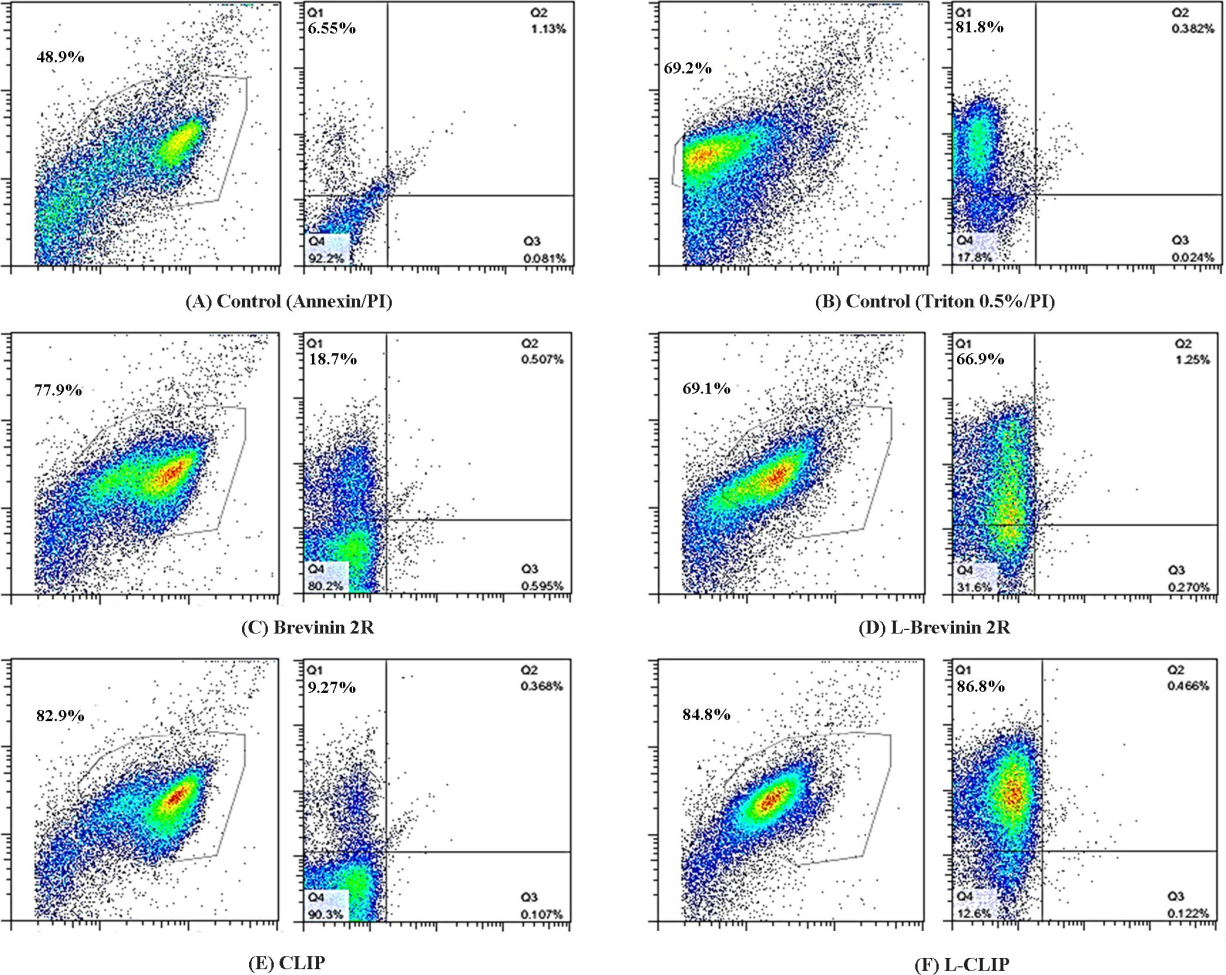
Flow cytometry results of *L*. *major* promastigotes exposed to different peptides. Effective peptides affected *L*. *major* through necrosis. No signs of apoptosis event were detected. (A) Untreated parasite stained with Annexin/PI, (B) Parasite treated with 0.5% Triton x-100 as positive control for PI. (C) Brevinin 2R, (D) L- Brevinin 2R, (E) CLIP and (F) L- CLIP treated parasite. In each pair of graphs, the left represents SSC vs FSC and the right graph indicates PI vs Annexin value.

To underlie the peptides’ mechanism of action on promastigotes, Annexin/PI assay was carried out on promastigotes treated with the peptides. Our findings exhibited that the population of necrotic *L*. *major* promastigotes increased from 6.55% in negative control to 66.9% and 86.8% in the promastigotes treated with L-Brevinin 2R and L-CLIP, respectively. Notably, Brevinin 2R and CLIP had 18.7% and 9.27% necrotic bodies (Fig 6).

### L- Brevinin 2R or Brevinin 2R did not activate caspase 3 and 7 in the promastigote

**Fig 7:**
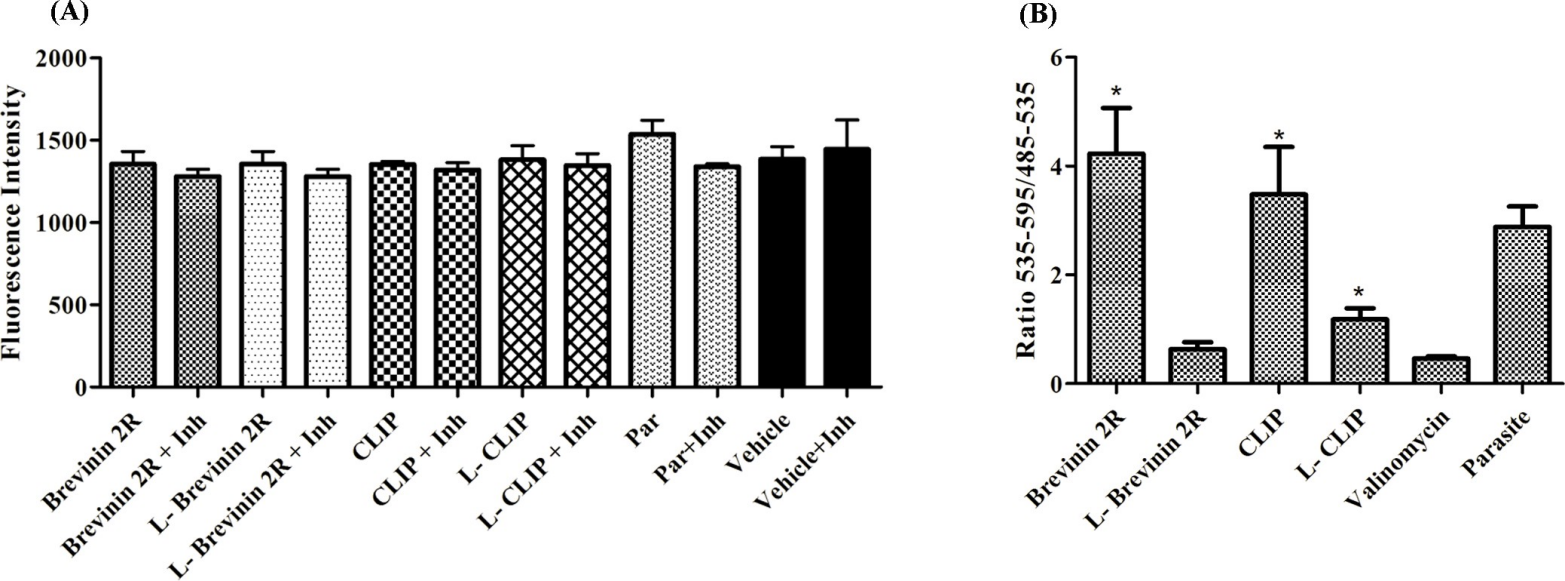
**(A)** Active caspase detection in *L*. *major* promastigotes exposed to peptides. Changes in the fluorescence intensity in the presence or absence of caspase inhibitors were studied. Inh: inhibitor. **(B)** Ratio of red to green fluorescence zone for mitochondrial electrical potential detection. Stars indicates the significant difference in compare to valinomycin. *, ** and *** = *P* value < 0.05, < 0.01 and < 0.001 respectively.

Apoptosis induction signs are detectable by tracking different key molecules involve in the process. In this regard, detecting active caspase 3 and 7 in the promastigote is a reliable method to find apoptosis [22]. In this method, using caspase substrate and determining the products indicates the presence of active caspases. No evidence of caspase activation was observed after Brevinin 2R or L- Brevinin 2R treatment in the promastigote. Also, promastigotes exposed to CLIP and L- CLIP did not show any sign of active caspase reaction. Furthermore, samples involved caspase inhibitor reagent (Ac-DEVD-CHO) displayed no diminished fluorescence signal in compare to the wells with caspase substrate alone. The fluorescence intensity in the samples was not significantly different with either the parasite alone or the media. These results imply that, apoptosis through caspase activation was not involved in these peptides’ mechanism of action (Fig 7A).

### Effect of L- Brevinin 2R on mitochondrial membrane potential

In order to find changes in the mitochondrial potential, the ratio of red to green fluorescence density of JC-1 probe in the promastigotes exposed to the peptides was measured. In the case of Brevinin 2R and CLIP, the ratio was far higher than one (4.2 ± 0.82 and 3.4 ± 0.87), indicating that JC-1 probe molecules accumulated and made large J- aggregate. On the other hand, L- Brevinin 2R had ratio lower than one (0.63 ± 0.13), which demonstrated that, JC-1 molecules are in monomeric form and mitochondria potential membrane was changed. By treating valinomycin as a positive control, a decreased amount of red to green ratio was seen (0.46 ± 0.03). According to Mann Whitney nonparametric test, Brevinin 2R and CLIP were significantly different with valinomycin in terms of red to green ratio which suggests that, there is no effect on mitochondrial potential as much as valinomycin. Additionally, L- CLIP affected mitochondria potential less strong than that of valinomycin or L- Brevinin 2R. Indeed, L- Brevinin 2R and valinomycin similarly disturbed the mitochondrial function (Fig 7B).

### Phenotypic changes of promastigotes exposed to L-Brevinin 2R and L-CLIP

**Fig 8:**
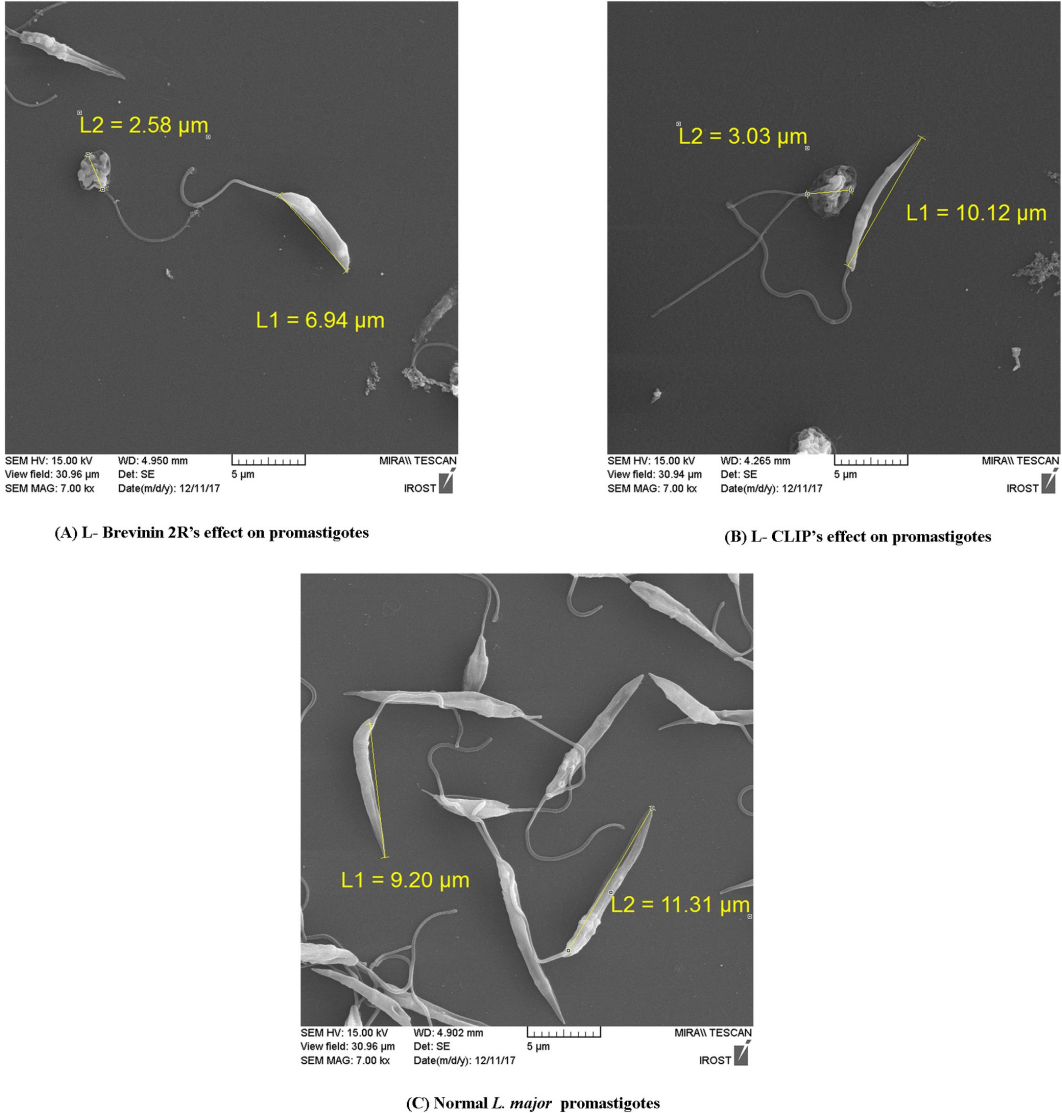
SEM view of promastigotes exposed to the IC_50_ concentration of L- Brevinin 2R (A), L- CLIP (B) and normal promastigotes (C). Pictures were captured with 7000X magnification.

Scanning electron microscopy pictures showed damaged promastigotes exposed to the IC_50_ concentration of L- Brevinin 2R and L- CLIP compared to the non- treated *L*. *major* promastigotes. Changes in the size of promastigotes were obvious in the parasites exposed to the effective peptides (L- Brevinin 2R, L- CLIP). Likewise, there was evidence of vesicle formation in the parasites or full deformity in their shape as compared with intact promastigotes (Fig 8).

### Site specific toxicity of L- Brevinin 2R in animal model

Among the peptides studied against *L*. *major*, L- Brevinin 2R was found to be the most effective one. Although L- Brevinin 2R showed toxic effect against THP1 and RBCs, it was selected for *in vivo* assay due to its effectiveness against promastigote stage at the low concentration. Moreover, it was the only peptide that could probably affect the amastigote form. Hence, we attempted to test its site specific toxic effect in the animal model before further evaluation for its efficacy. During the three weeks follow up, other than one mouse with a black toe in the group received 30 μg of peptide, we couldn’t find any signs of tenderness, uneasy walking, or painful footpad in the studied Balb/c mice. Thus, we chose the lower tested concentration (20 μg per mouse) for further experiments (S1 Fig).

### Administration of CpG motif in combination with peptide in treatment protocol

It was known that, CpG motif application would be helpful to conduct a specific immune response in experimental models [23,24,25].

In previous studies, it has been shown that, combination of CpG motif with LL-37 peptide could induce better therapeutic effect against ovarian cancer in C57BL6 mice [26]. Due to the importance of having a Th1 response in *Leishmania* infection, we used CpG in combination with peptide treatment (Table 4).

**Table 4:** Treatment protocol in experimental animal model.

Groups	Amount and rout of injection	Number of injections	Duration of treatment
**G1 (Peptide + CpG)**	Peptide:20 μg/mouse (s.c) CpG:45 μg/mouse (i.p)	Peptide: 5 times CpG: Once	10 days
**G2 (Peptide)**	20 μg/mouse (s.c)	5 times	10 days
**G3 (DMSO)**	50 μl (s.c)	5 times	10 days
**G4 (PBS)**	50 μl (i.p)	10 times	10 days
**G5 (AmB)**	8 mg/kg (i.p)	10 times	10 days

### An increase in footpad swelling following L-Brevinin 2R administration

**Fig 9:**
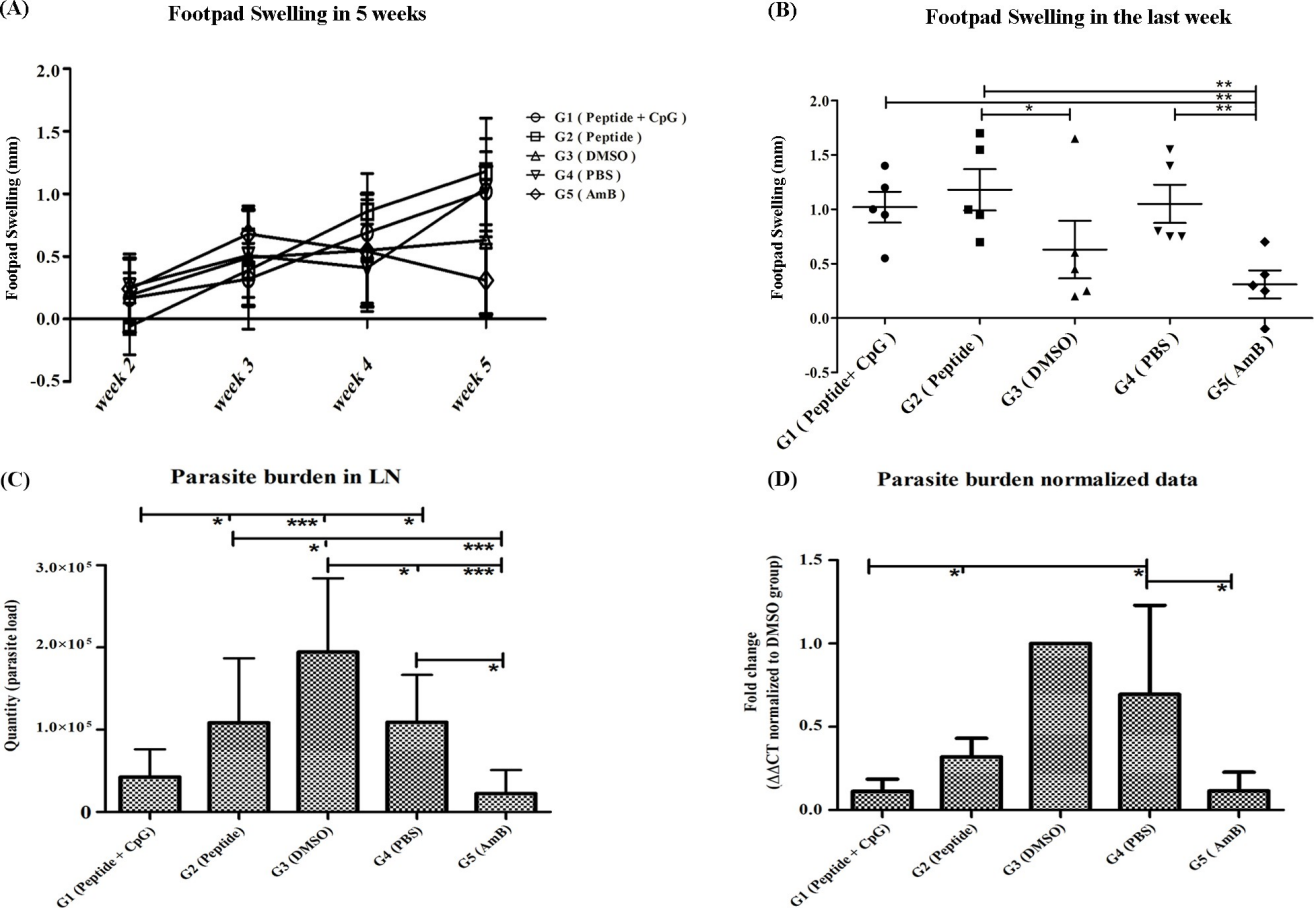
**(A)** Footpad swelling in weeks after challenge until the end of treatment. Swelling was calculated according to thickness and width of the footpad. Infected foot size was subtracted from intact footpad. (**B**) Footpad swelling in experimental groups at 5^th^ week after challenge. **(C)** Real-time PCR assay for parasite burden determination in the lymph node of experimental animals. Peptide (L- Brevinin 2R) with or without CpG motif was able to control the parasite load in the lymph nodes adjacent to the infected footpad. (D) Normalized data provided from GAPDH house-keeping gene Real- time PCR assay in parallel with parasite burden in lymph node samples. *, ** and *** = *P* value < 0.05, < 0.01 and < 0.001 respectively.

In cutaneous leishmaniasis experimental model of Balb/c mice, footpad swelling occurs after infectious challenge [27]. Therefore, we expected that, following treatment the size of swelling in the site of infection would be decreased. Surprisingly, in the fifth week after challenging, footpad swelling in group 1 (peptide + CpG) showed a significant increase in compare to group 5 (AmB). Furthermore, footpad swelling in group 2 (Peptide) significantly increased compared to group 3 (DMSO) as well as group 5 (AmB). Notably, AmB could significantly control the footpad swelling in group 5 compared to PBS (group 4). Collectively, the results demonstrated that, application of L- Brevinin 2R not only did not reduce the footpad swelling but also increased the size of footpad in the last week of treatment (Fig 9A and 9B).

### Parasite load decreased in the groups treated with L- Brevinin 2R

Parasite quantity was measured using absolute copy number and relative burden using Real-time PCR according to a standard curve and GAPDH normalization in popliteal infected lymph nodes. Group1 (Peptide + CpG) showed a significant decrease (*P*<0.05) in the parasite load in compare to peptide alone group (group 2), group 3 (DMSO) (*P*<0.001) and group 4 (PBS) (*P*<0.05) as well. Reduction of parasite load in the group 1 (Peptide + CpG) compared to peptide alone (group 2) indicated that, the presence of CpG motif had positive effect on controlling the infection. Also, AmB showed a significant decrease in the parasite load in compare to DMSO (group 3) and PBS (group 4) (*P*<0.001). Moreover, a significant difference was observed between group 2, the mice treated with peptide alone, and group 3 (DMSO) (*P*<0.05). Moreover, the normalized data with GAPDH gene showed that, group 1 (Peptide + CpG) had significant difference (*P*<0.05) with the Peptide only group. Furthermore, the group treated with AmB had significant difference (*P*<0.05) with group 4 (PBS). These findings implied that, peptide application could successfully inhibit and control the parasite proliferation in the popliteal lymph nodes adjacent to the infection site (Fig 9C and 9D).

### Cytokine production profile in experimental animal model in favor of Th1 response is controversial

**Fig 10:**
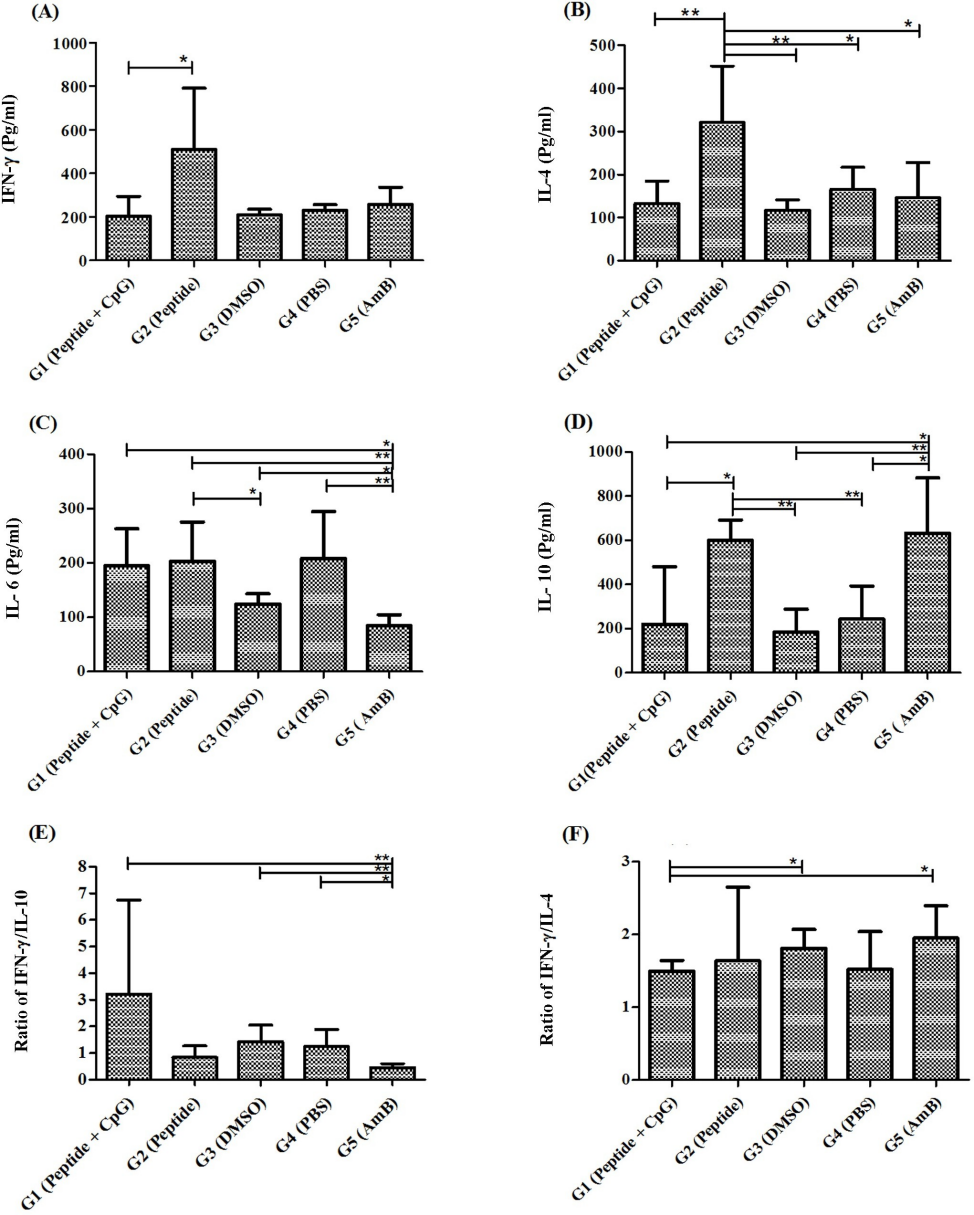
Cytokine production profile (A) IFN-γ, (B) IL-4, (C) IL-6, (D) IL-10, (E) IFN-γ/IL-10 ratio and (F) IFN-γ/IL-4 ratio in splenocytes exposed to freezed-thawed antigen of *L*. *major*. *, ** and *** = *P* value < 0.05, < 0.01 and < 0.001 respectively.

In the group that peptide alone was administered (group 2), the level of IFN-γ production was significantly higher than that treated by Peptide + CpG. No significant difference was found between group 1 and control group in terms of IFN-γ production. No elevated amount of IFN-γ was also detected in the AmB treated group (Fig 10A). The mice treated with the peptide (group 2) produced higher level of IL-4 than all the other groups (Fig 10B). Additionally, IL-6 production in groups 1, 2, 3, 4 were significantly higher than the group 5 in which mice were treated with AmB (group 5) (Fig 10C). Furthermore, AmB treated group showed a higher amount of IL-10 production in compare to the group administered Peptide + CpG (group 1), DMSO (group 3) and PBS (group 4). Remarkably, the level of IL-10 in group 2 (Peptide), was significantly higher than that of group 1 (Peptide + CpG), group 3 (DMSO) and group 4 (PBS) (Fig 10D). Noteworthy is that, the ratio of IFN-γ to IL-10 in group 1 was higher than other groups and was significant to AmB (Fig 10E). Also, this ratio was lower in in AmB treated mice in compare to PBS and DMSO groups. The amount of IFN-γ to IL-4 was significantly higher in AmB in compare to group 1 (Peptide + CpG) (Fig 10F).

## Discussion

Treatment of leishmaniasis is a serious problem due to the increasing resistance against current drugs, toxicity, lifelong side effects and high cost of medications. Hence, designing new drugs against leishmaniasis are in urgent need. Nowadays, antimicrobial peptides as new generation of natural and synthetic antibiotics are widely tested against bacterial and fungal infections. Some are currently in different phases of clinical trials such as Brilacidin, for oral mucositis treatment in phase II or Pexiganan for diabetic foot ulcers and Surotomycin for *Clostridium difficile* infections in phase III [28] or even in the market like PL-5 for bacterial skin infection [29]. Brevinin 2R, a peptide isolated from frog skin secretion, showed antifungal, antibacterial and anti-cancer effects [17]. Non- hemolytic features of Brevinin 2R, highlights it as an ideal drug candidate against infectious agents [18].

In the current study, anti-leishmanial effect of Brevinin 2R and its fatty acid conjugate version was investigated against promastigote as well as amastigote forms of *L*. *major*. Furthermore, the mechanism of action of this peptide was studied against promastigotes. After evaluating its toxicity effect in the experimental animal, therapeutic potential of L- Brevinin 2R was also tested against cutaneous infection in Balb/c mice. Our findings revealed that, conjugation of lauric acid to the peptide enhanced its antibacterial and anti-leishmanial effects. Also, this novel conjugated version resulted in increased toxicity against RBCs and THP1 cells. Interestingly, lauric acid conjugation affected CLIP specificity and converted it to an antibacterial and anti-leishmanial agent. Surprisingly, lauric acid alone did not possess the same effect either on the bacteria or parasite. This evidence demonstrated that, the conjugated peptide following addition of lauric acid, enabled it to easily access to the membrane and inside the cell, although the physical characteristics of this novel conjugated peptide needs to be further studied. The mechanism underlie for the antimicrobial peptides is through two main pathways: membrane disruption or triggering vital functions inside the cell leading to either apoptosis or cell death [12]. In our study, L- Brevinin 2R limited promastigote proliferation through increasing the membrane permeability and changing the membrane as well as mitochondrial potential. Consistent with these data, SEM analysis also showed considerable changes in the size and shape of promastigotes, which were exposed to either L- Brevinin 2R or L- CLIP. Furthermore, large vesicle formation in the membrane revealed that, promastigotes were at the brink of death.

Accumulating evidence demonstrated that, Brevinin 2R was able to inhibit cancerous cell (Jutkat, L929 and MCF-7) growth mainly by autophagy; however, apoptosis induction was also reported by this peptide [17]. In this regard, Brevinin 2R and its derivatives were able to limit breast adenocarcinoma cells (MCF-7) and lung carcinoma cells (A549) proliferation by apoptosis induction [30]. In the present study, no sign of apoptosis induction was seen in the promastigotes exposed to Brevinin 2R or lauric acid conjugated form of the peptide. Caspase activation was also not detected in the promastigote culture and necrosis was the only incident happened to the peptide treated promastigotes. Moreover, Phosphatidylserine out casting from the cell membrane as the most obvious signature of apoptotic cells was not detected in our assay. It is crucial to mention that, peptide’s mechanism of action on promastigotes could not be extrapolated to amastigote stage of the parasite.

On the other hand, our target peptide L- Brevinin 2R, showed toxicity against THP1 cells; however, no site specific adverse effect was observed in our *in vivo* experiment in Balb/c mice at the concentrations lower than 30 μg. Moreover, the therapeutic potential of L- Brevinin 2R in *L*. *major* infected Balb/c mice was investigated in our study. The results displayed that, L- Brevinin 2R plus CpG motif or L- Brevinin 2R alone were not able to control footpad swelling but, in both groups parasite load was significantly lower than the control. This finding suggests that lesion size is not necessarily correlates with parasite load in the tissue [31]. Furthermore, we did not know the exact effect of CpG motif in this regard, meanwhile we hypothesize that, the presence of CpG motif could be helpful in activation of immune responses on behalf of healing process. In a research by Chuang *et al*. presence of LL37, an antimicrobial peptide from cathelicidin family could increase CpG motif uptake inside the cells to access easier to endosomal TLR9. Also, they reported that NK cell proliferation was increased in combination therapy with the peptide and CpG [26].

Leishmaniasis healing process is due to overcoming the Th1 responses to Th2 profile. In this regard, IFN-γ and TNF-α are the important cytokines in the induction of iNOS, induced nitric oxide synthase, leading to the respiratory burst in macrophages and subsequently intracellular limitation of amastigote growth inside the macrophages. Presence of the high levels of IL-10 and IL-4 in the infected mice indicates the non-healing *Leishmania* infection [32]. IL-6; a pro inflammatory cytokine, also induces IL-17 expression by Th17 regulatory cells which in turn, resolves intracellular infections. On the other hand, IL-10 can down regulate IL-17 production in the immune cells separated from CL (Cutaneous leishmaniasis) and ML (mucocutaneous leishmaniasis) patients [33]. IL-4 quenches the activated macrophages and promotes Th2 responses in leishmaniasis [34]. Hence, increased IFN-γ and IL-6 levels as well as decreased amount of IL-10 and IL-4 skewed to the healing process in leishmaniasis. In this study, we expected an unpredictable change in the production of the cytokines’ profile due to the immunomodulatory effect of antimicrobial peptide and CpG motif which was applied in the experiment. In line with this hypothesis, Ferrante A. *et al*. demonstrated that, AmB application in the mice, suppressed the immunological responses. Also, the drug was able to reduce the DTH reaction [35]. In the other study, it was proved that, AmB reduced different cytokine (IFN-γ, IL-4, IL-10 and IL-12) production in *L*. *major* infected Balb/c mice in compare to control, right after the end of drug treatment course [36]. Accordingly in the current study, in AmB group (group 5), the level of IFN-γ was the same as the other groups. Likewise, production of IFN-γ in Peptide + CpG (group 1) or Peptide treated group (group 2) did not show any significant difference compared to the control or AmB group. Previously, it has been shown that, Brevinin 2R up regulated the IL-6 expression in HepG2 cells in *in vitro* condition [19]. In this research, it was revealed that application of L- Brevinin 2R in Balb/c (group 2) significantly increased IL-6 production in compare to vehicle control (DMSO) in the stimulated splenocytes. On the other hand, AmB caused a significant suppression in IL-6 production compared to all the other groups which was not unexpected. The level of IL-4 in the peptide treated group, was higher than that the other groups. Moreover, the ratio of IFN-γ to IL-4 did not support healing process. Meanwhile, the ratio of IFN-γ to IL-10, in group 1 (Peptide + CpG) showed promising results which is associated with the reduced parasite burden observed in this group. Although the ultimate and the most important sign of healing is to control the parasite load in the tissue, still other parameters such as production of cytokines and chemokines are more difficult and complex especially for antimicrobial peptides that have strong immunomodulatory effects through various pathways.

In conclusion, we demonstrated that, L- Brevinin 2R limited and controlled proliferation of the promastigotes and probably amastigotes of *L*. *major* in an *in vitro* model. Membrane disruption, changes in the membrane and mitochondria potential were the underlying mechanisms in which the peptides act on promastigote form. Although the cytokine profile production and footpad swelling data in the peptide treated groups were controversial, parasite burden depletion in the peptide treated groups (groups 1 and 2) showed a partial resolution of infection in highly susceptible Balb/c mice model. Our study revealed that, antimicrobial peptides with correct modifications may serve as novel drug candidates against infectious agents.

## Materials and methods

### Reagents and chemicals

Sytox green and bis-(1,3-diethylthiobarbituric) trimethine oxonol (bisoxonol) were purchased from Molecular probes. Fetal calf serum (FCS) was from Gibco. RPMI, DMEM, M199, HEPES, L- glutamine, hemin, adenosine, lectin, valinomycine, NaCl, yeast extract, lauric acid, concanavalin A, sodium dodecyl sulphate (SDS) and ficoll were prepared from Sigma. Trypton was from Difco and amphotericin B (AmB) was purchased from Cipla, India. Alamar blue from Invitrogen, draq5 was from Biostatus. Paraformaldehyde was from Fisher, DMSO was from Daejung. Glutaraldehyde, sodium cacodilate and BSA (bovine serum albumin) were from Merck.

### Peptides

Peptides (Brevinin 2R, lauric acid- Brevinin 2R, CLIP (MHC Class II associated invariant chain peptide) and lauric acid- CLIP) were synthesized by Biomatik (Cambridge, Canada) in trifluoroacetate (TFA) salt and purified by HPLC with water and acetonitrile with > 95% purity. The molecular mass of synthesized products was confirmed with mass spectrometry report, provided by manufacturer.

**Parasite and animals** *L*. *major* promastigotes (MRHO/IR/75/ER) were cultured in M199 media supplemented with 10% FCS, 4 mM HEPES, 2 μM L- glutamine, 0.3μM hemin, 10 mM adenosine, 0.7% V/V gentamycin, and incubated at 26°C for five days. Metacyclic promastigotes were prepared through ficoll gradient centrifugation followed by washing with PBS (phosphate buffer saline, 137 mM NaCl (Sigma), 0.27 mM KCl (Merck), 1.5 mM KH_2_PO_4_ (Merck), 8.1 mM Na_2_HPO4). The total of 2x10^6^ *L*. *major* metacyclic promastigotes was injected subcutaneously in the hind footpad of Balb/c mice for infectious challenge.

Freezed—thawed antigens of parasite were prepared from 10^8^ logarithmic phase *L*. *major* promastigotes with sequential freezing in liquid nitrogen and thawing at 37°C water bath. Protein concentration was approved with BCA kit (Thermo Scientific) according to manufacturer’s instruction.

Balb/c mice with 6–8 weeks of age were purchased from breeding stock of Pasteur Institute of Iran in Karaj. All animals were hosted, fed and humanly killed according to standard institutional guidelines.

### Ethical concerns

The checklist of standard condition for manipulating animals were prepared and handed to the ethical committee in Pasteur Institute of Iran. Experimental condition, treatment and euthanizing protocols were subjected to deep investigation by ethical committee and ultimately confirmed and approved with the code of 95/0110/138l00 in Pasteur Institute of Iran. The checklist was based on the Specific National Ethical Guidelines for Biochemical Research issued in 2005 by the Research and Technology Deputy of Ministry of Health and Medicinal Education (MOHM) of Iran. Also, regards to the part of the experiment which has been performed in Pasteur Institute of Korea (*in vivo* site specific toxicity effect in Balb/c mice), local ethical committee approved the protocols according to their institutional policy (ethical code number: IPK-17008)

### Peptides’ effect on *E*. *coli* bacteria (ATCC 25922)

An overnight starter culture of *E*. *coli* was applied to prepare at 1:1000 dilution of bacteria in LB medium (0.5% w/v yeast extract, 1% w/v trypton, 1% w/v NaCl). Incubation was continued in 37°C shaking incubator (Inforce HT Bottmingen) till O.D (optical density) of culture reached to 0.4–0.5 in 600 nm of wavelength. Different volumes of bacteria were diluted with PBS 1x to find the O.D equal to 0.003. This stock was used to prepare sequential one tenth dilutions (10^−1^ to 10^−6^) of culture and seeded on LB agar (LB medium + 0.02% w/v agar) solid plate to find out the best dilution which leads to about 150 to 200 countable bacterial colonies. The best dilution was selected for further studies with peptides (2x10^-3^ dilution). Equal volumes of bacteria and different dilutions of either peptides (6.25, 3.125 and 1.562 μg/ml) or lauric acid, were co cultured in sterile 96 wells plate for 3 hours in 37°C shaking incubator. Then, each well transferred to a LB agar plate and incubated overnight. The day after, bacterial colonies were counted. All experiments compared with a negative (PBS 1x) and positive control (ampicillin) also, the experiment was performed in duplicate. The experiment repeated three times for confirmation. According to negative control colony count, percentage of viable remaining bacteria was calculated [37].

### Peptides’ effect on *L*. *major* promastigotes

Two days old, logarithmic phase promastigotes were harvested and seeded in 96 well plates (2x10^6^ cell/well). Peptides were added to the parasites from 500 to 10 μg/ml in duplicate. AmB was used as a positive control drug (5 μ/ml, 10 dilutions, 1:2) and incubated at 26°C for 24 hours. The day after, alamar blue reagent was added as 1:10 volume of each well and incubated for additional 4 hours in the dark. Plate absorbance was measured at 570 and 600 nm in microplate reader (Epoch, BioTek, USA). Percent of viability was calculated according to manufacturer’s instruction in alamar blue reagent manual [38]. Also kinetic study of peptides’ function against *L*. *major* promastigotes has performed at 24, 48 and 72 hours after exposure.

### Peptides’ effect on *L*. *major* Amastigotes and THP1 cell

An image based assay applied to find out the effect of peptides against THP1 cells (ATCC TIB-202) and its internalized form of *Leishmania* which transforms to amastigote inside the cell. PMA (Phorbol 12-Myristate 13-Acetate, 50 ng/ml) treated THP1 cells (0.8x10^4^ cell/well) were seeded in 384 wells black plate (Greiner) and incubated at 37°C CO_2_ incubator for 48 hours. Lectin isolated (50 μg/ml) stationary phase promastigotes of *L*. *major* (1.6x10^5^cells/well) were added to THP1 cells and incubated for another 24 hours [39]. Lectin isolation of promastigotes was in the following order: live parasites were separated with centrifugation, washing process was performed two times with PBS. Cells were diluted to 10^8^ cell/ml in PBS, after addition of lectin (50 μg/ml), incubation was performed for 30 min in 28°C shaking incubator (Jeio Tech, South Korea). Then, metacyclic promastigotes were isolated by centrifugation from non-precipitated cells in supernatant. Plate was washed with PBS and dissolved drugs in 2% RPMI and 1% DMSO were added. Peptides were diluted from 250 to 0.48 μg/ml and AmB was diluted from 25 to 0.048 μM, also high concentration of AmB was applied as control. Moreover, untreated infected THP1 cells considered as negative control. Incubation continued for another 24 hours. The day after, fixation with 16% paraformaldehyde (30 min incubation at RT) was performed, followed by 5 times washing with PBS. Draq5 fluorescence probe was added (1:1000) in 4% paraformaldehyde for 3 hours and imaging the plate with Operetta High Content Screening System (Perkin Elmer) imaging device was done with 635 nm filter. Four pictures were captured from each well. Pictures were analyzed and quantified with Image mining software (software developed by Pasteur Institute of Korea). Data normalization was done according to negative control (1% DMSO) and positive control (AmB). Then, normalized number of total cells, total parasite and infected cells were calculated [40].

### Hemolysis assay

Red blood cells (RBCs, provided by Iranian Blood Transfusion Organization) were isolated from 3 ml fresh human blood by centrifugation. RBCs were washed with PBS (3 times) and diluted to 20 ml. 180 μl of diluted RBCs was exposed to either peptides’ dilution (500–25 μg/ml), Triton x-100 0.01% as positive control or PBS, as negative control in a 96 well U-shaped plate (Greiner) and incubated at 37°C for 30 min. The plate centrifuged and supernatant’s optical density was measured with a microplate reader (Tecan, USA) in the wavelength of 540 nm. Percent of hemolysis was calculated with the following formula [41]:

$$\text{\%Hemolysis}=\frac{\lambda \;\text{Test}-\lambda \;\text{negative control}}{\lambda \;\text{positive control}}\text{x}100$$

### Assaying peptides’ function on the membrane of promastigote

The effect of peptides’ on promastigote membrane was persuaded by Sytox green fluorescence probe. The dye is a DNA binding molecule with bulky structure which passes through the membrane if large pores are existed [42]. Two days old logarithmic phase *L*. *major* promastigotes were harvested, washed and seeded in a 96 well black plate (BRAND plates (2x10^6^ cell/well). Sytox green probe was added to each well (1μM) in equal volume with the parasite and incubated for 15 min in dark, then fluorescence intensity was measured using microplate reader (Cytation 3, BioTek, USA) with excitation filter: 485 nm and emission filter: 520 nm, every 10 min. After two times of measurement, samples; either peptides in appropriate dilution, or 0.1% Triton x-100 or media were added to each well. Fluorescence intensity measurement continued right after addition of reagents and repeated every 10 minutes up to70 min. Then, increase in the fluorescence intensity was calculated from the ratio of every sample’s intensity to negative control (parasite + Sytox green).

### Assaying peptides’ function on membrane electrical potential

Bis-(1,3-diethylthiobarbituric) trimethine oxonol (bisoxonol) probe (0.2 μM in HBS buffer containing 21mM HEPES, 0.7mM Na_2_HPO_4_, 137mM NaCl, 5mM KCl. 6mM D-Glucose) was added to each well of 96 well black plate (BRAND plates) containing the same volume of 2x10^6^ of mid log phase *L*. *major* promastigotes. Fluorescence intensity was measured with microplate reader (Cytation 3, BioTek, USA) once. After that, peptides in appropriate dilution or media only were added. Fluorescence intensity measurement continued right after addition of the reagents and followed every ten minutes up to 80 min.

### Apoptosis or necrosis induction in the promastigotes

Two days old *L*. *major* promastigotes (2x10^6^ cell/ml) were exposed to IC_50_ amount of peptides in 96 well plate (Greiner) and incubated at 26°C for 24 hours. The day after, each well was harvested by centrifugation and washed with PBS 1x once. According to Annexin V-FITC (Biovision kit, USA), cells were diluted in Binding Buffer and Annexin V was added (5μl) after 5 min incubation at room temperature, then PI was added (5μl) and amount of apoptotic or necrotic cells were determined with flow cytometry (Partec, Sysmex, Germany). Untreated parasites were also exposed to Annexin V, PI or both as controls. 0.5% Triton was also applied as positive control for PI. The results were analyzed by considering the viable percentile (Annexin V -/PI-), early apoptotic (Annexin V +/PI-), late apoptotic (Annexin V +/ PI +) and necrotic (Annexin V-/PI+). The flow cytometry data were analyzed with Flow Jo software (Three star, version 7.5). On FSC / SSC plots the gates were applied to exclude debris.

### Caspase 3 & 7 activation in promastigotes

5x10^5^ two days old *L*. *major* promastigotes were seeded in a 96 well black plate (Greiner). Parasites were exposed to IC_50_ concentration of peptides for 24 hours. After cell precipitation, supernatant was aspirated thoroughly and caspase substrate (Apo-ONE Hoemogeneous caspase-3/7 Assay kit (Promega)) or substrate plus inhibitor (Ac-DEVD-CHO, Biomol,10μM) were added to separated wells and incubated overnight. Rhodamine 110 production as the result of active caspase presence was measured in green wavelength (Exc = 499 nm—Emi = 521 nm, SPEKTRAmax M5, Molecular Devices). Parasite only and PBS 1x also were applied as negative controls.

### Mitochondrial potential (Δψ_M_) changes

JC-1 lipophilic cationic fluorescence probe (Cayman Chemicals) was applied for assaying potential changes in the mitochondria. JC-1 can enter to the mitochondria and change the fluorescent intensity according to its monomeric or aggregated structures. In the intact cells with healthy mitochondria, JC-1 molecules make huge complexes as J-aggregates with fluorescence emission in the red zone of spectrum. On the other hand, in the injured mitochondria, JC-1 probe is monomeric molecules with intense green fluorescence. The ratios of red to green intensity visualize mitochondrial wellness or injury. IC_50_ concentration of peptides were added to 5x10^5^ two days old *L*. *major* promastigotes in a 96 well black plate (Greiner) and incubated at 26°C for 24 hours. 1:10 dilution of JC-1 probe with RPMI were added to each well and incubated for 30 min. After precipitation of the cells, supernatant was substituted with JC-1 buffer and the procedure was repeated two times before measuring the fluorescence intensity in red with a excitation wave length of 535 nm and emission of 590 nm and green (excitation of 485 nm and emission of 535 nm) area of spectrum (SPEKTRAmax M5, Molecular devices). The ratio of red to green indicated mitochondrial potential situation. Valinomycin (0.5 μM) was applied as positive control which was added 30 min before the test has been started and media alone as negative control.

### Scanning electron microscopy (SEM) analysis

In order to study changes in the appearance of promastigotes exposed to peptides, Scanning Electron Microscopy (SEM) features were prepared. Briefly, 2x10^6^ mid log phase *L*. *major* promastigotes were exposed to IC_50_ concentrations of effective peptides (L- Brevinin 2R, L- CLIP) for 24 hours. After washing with PBS, samples were fixed with 0.5% Glutaraldehyde then, Sodium cacodylate (0.1 M) was applied for extra washing stage and fixation completed with 1% Tetroxide osmium. Dehydration process was performed using different concentrations of ethanol (25%, 50%, 70%, 90% and 100%), after that the samples dried in desiccator overnight. Samples were coated with gold and studied using SEM system (TESCAN, MIRA II LMU, FIELD EMISSION).

### Site specific toxicity assay in Balb/c mice

8 groups of 6–8 weeks Balb/c mice were injected subcutaneously in the left hind footpad with different dosage of L- Brevinin 2R (30, 20, 8, 4, 1.6, 0.8, 0.4 μg), and water + DMSO only (as control) weekly for three weeks. Footpad swelling, tenderness or inflammation in the footpads were observed carefully every week up to 5 weeks from incidence of the test.

### Treatment protocol in *L*. *major* infected animals

Five groups of Balb/c mice (n = 5) were infected with 2x10^6^ metacyclic stationary phase *L*. *major* promastigotes in the left footpad. Four weeks after infection, treatment was initiated. Group1, treated with 20 μg/mouse of L- Brevinin 2R (s.c) every other day and CpG motif, as a single i.p injection (45μg/mouse). Group 2 received L- Brevinin 2R alone exactly as the first group. Group 3 received vehicle (DMSO + Water) (s.c). Group 4 received PBS and group 5, treated with AmB (8 mg/kg) as i.p injection every day for ten days sequentially.

### Footpad swelling measurement and animals’ body weight

Footpad size was measured with a metric caliper after infectious challenge. Footpad swelling was calculated through subtracting infected foot from intact one.

Footpad swelling=(Height+Width)/2

### Parasite load in the lymph node

Immediately after finishing the treatment, mice were euthanized and popliteal lymph node from infected foot was exited and stored in PBS 1x in -20°C freezer. Each lymph node grinded by a sterile pellet pestle in PBS 1x, then, the sample was applied for genomic DNA extraction (GF-1 Blood and tissue extraction kit, Vivantis, Malaysia). First BB buffer and proteinase K were added to the grinded lymph node before incubation at 65°C for 30 min in dry block (Techne, Germany). After addition of pure ethanol, the mixture transferred to the spin column and centrifuged at 5000g/1min. Three stages of washing and centrifugation were performed using two different buffers. Ultimately, genomic DNA was extracted with distilled water. Absolute mode of Real-time PCR with a standard curve was applied for parasite burden quantification. To prepare standard sample, genomic DNA of 2x10^7^ stationary phase *L*. *major* promastigotes were applied with six serial dilutions (1:10). Real-time PCR master mix was prepared according to the Quantifast SYBR Green Real-time PCR kit (Qiagen, Germany). Briefly, 5 pmols of Primers, RV1 (forward: 5'-CTTTTCTGGTCCCGCGGGTAGG-3') and RV2 (reverse: 5'-CCACCTG GCCTATTTTACACCA-3') were used to amplify genomics. In parallel, the data was normalized to the mammalian house-keeping gene glyceraldehyde 3-phosphate dehydrogenase (GAPDH). GAPDH primers (Forward: 5’CGTCCCGTAGACAAAATGGT3’; Reverse: 5’TTGATGGCAACAATCTTCAC3’) (500 nM) were applied to perform reaction. Syber green (1 μM) and Rox (1:200 dilution) as reference dye were used with 15 ng of each genomic DNA in reaction. Amplification program was performed in Applied Biosystem 7500 as 95°C/ 10 min, then 95°C/15s, 60°C /30s and 72°C/ 40s (40 cycles). Melting and amplification plot prepared automatically by software and parasite quantity of each sample determined in compare to standard curve equation. Also, for data normalization, ΔCT of samples were calculated from subtracting each samples CT from the same on GAPDH. Then, ΔΔCT was obtained from subtraction of samples ΔCT from the vehicle (Group 3 (DMSO)). Finally, fold change was calculated according to the 2^-ΔΔCT^ formula.

### Cytokine assay in spleen

The day after the last treatment, 3 mice from each group were sacrificed and spleens were exited and grinded in sterile condition. RBCs were objected to complete lyses with ACK lyses buffer (NH_4_Cl 0.15M, KHCO_3_ 1mM, Na_2_EDTA 0.1 mM) for 5 minutes. After two times washing with media, cells were counted and seeded in 48 well sterile culture plates (5x10^6^ cell/ml) (Greiner). Splenocytes were exposed to either freezed–thawed antigen of *L*. *major* promastigotes (15 μg/ml), media only as negative control or Con A (concanavalin A, 5μg/ml) as positive control and the plates preserved in humidified 37°C CO_2_ incubator. Cell supernatants were collected in appropriate time after incubation (one day later for IL– 6, three days for IL– 4 and five days for IL– 10 and IFN- γ). Sandwich ELISA test was performed according to Duo Set kit (R&D System, USA) instructions. Briefly, capture antibody was coated in 96 well high binding plates (Greiner) overnight. After washing, wells were blocked with BSA for an hour and standards’ serial dilution or samples were added to the plate and incubated following another washing stage. Then, detection antibody was added and incubated for 2 hours. Streptavidin conjugated horse radish proxidase was applied to complete the reaction. Color reaction appeared with substrate (KPL, ABTS) addition. At last, the absorbance was measured by a microplate reader (TECAN, USA) at the wavelength of 405 nm.

### Statistical analysis

All data were demonstrated as mean ± SD and graphed using Graph pad prism version 5. Statistical analysis was performed with non-parametric Mann-Whitney test and two way ANOVA with *P < 0*.*05*.

## Supporting information

S1 Fig (A) Footpad swelling in mice groups after administration of different dosages of L-Brevinin 2R. Groups 1 to 8 received 30, 20, 8, 4, 1.6, 0.8, 0.4 μg, and water + DMSO respectively. (B) Photo images of mice received L-Brevinin 2R in site specific toxicity assay.
(TIF)
