# Correction: Anti-leishmanial activity of Brevinin 2R and its Lauric acid conjugate type against *L*. *major*: *In vitro* mechanism of actions and *in vivo* treatment potentials

**DOI:** 10.1371/journal.pntd.0007584

**Published:** 2019-07-16

**Authors:** Farnaz Zahedifard, Hyeryon Lee, Joo Hwan No, Mona Salimi, Negar Seyed, Ahmad Asoodeh, Sima Rafati

There are errors in the Funding statement. The correct Funding statement is as follows: The current study is funded by Iran National Science Foundation with grant ID 940007 and 95824986 to Sima Rafati. Also Farnaz Zahedifard received funding from Pasteur Institute of Iran as part of her Ph.D studentship. She also got Calmette and Yersin training scholarship from RIIP foundation from Institut Pasteur International Network for her training period in South Korea. The founders had no role in study design, data collection and analysis, decision to publish or preparation of the manuscript.
