# Supplementary figures and images for: Anti-leishmanial activity of Brevinin 2R and its Lauric acid conjugate type against L. major: In vitro mechanism of actions and in vivo treatment potentials
Source: PLoS Negl Trop Dis. 2019 Feb 27;13(2):e0007217. doi: 10.1371/journal.pntd.0007217 (PMC6411200; doi:10.1371/journal.pntd.0007217)

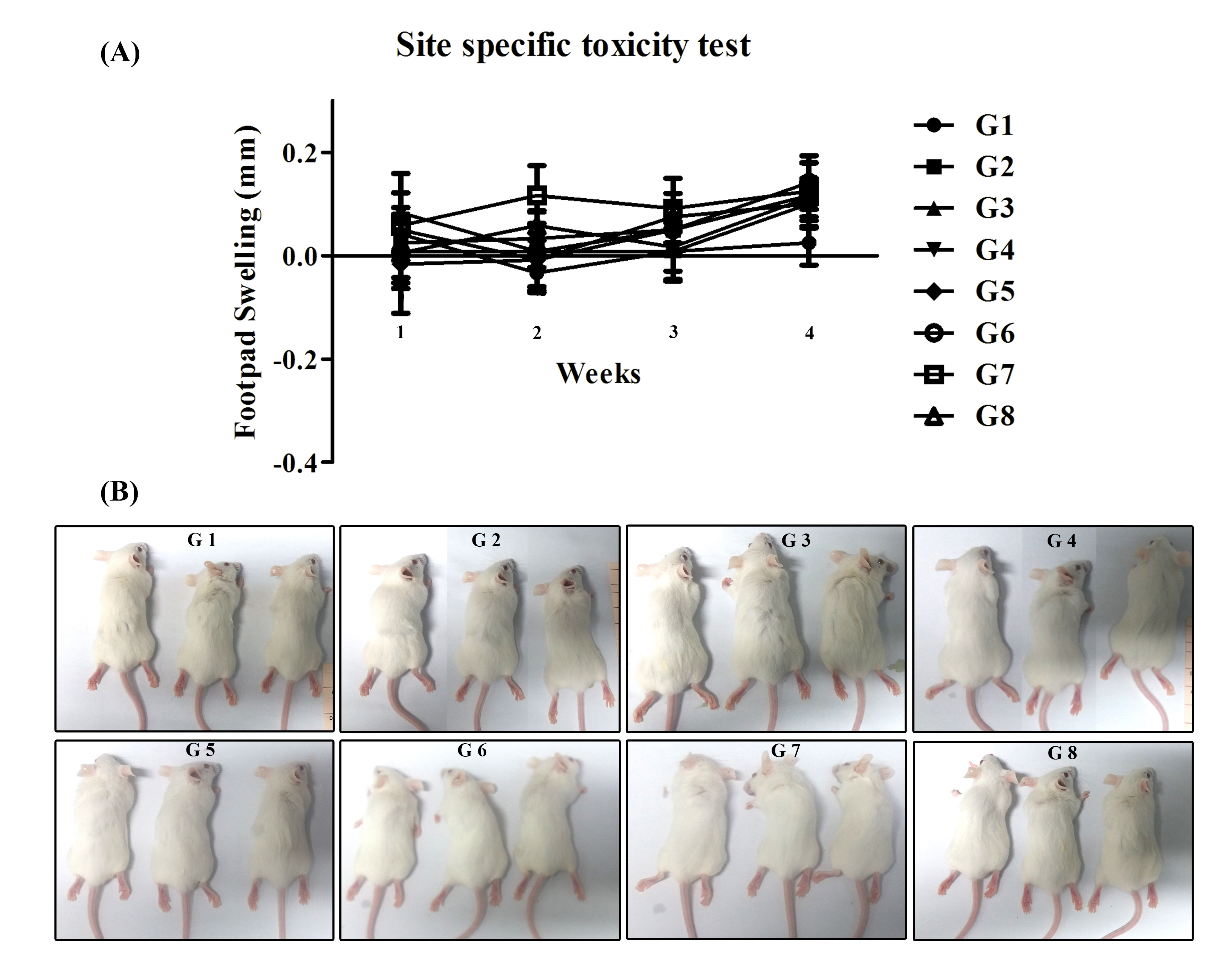

Supplement: S1 Fig — (A) Footpad swelling in mice groups after administration of different dosages of L-Brevinin 2R. Groups 1 to 8 received 30, 20, 8, 4, 1.6, 0.8, 0.4 μg, and water + DMSO respectively. (B) Photo images of mice received L-Brevinin 2R in site specific toxicity assay. (TIF) [file pntd.0007217.s001.tif]
